# Circulating tumor DNA mutation profile is associated with the prognosis and treatment response of Chinese patients with newly diagnosed diffuse large B-cell lymphoma

**DOI:** 10.3389/fonc.2022.1003957

**Published:** 2022-11-17

**Authors:** Tao Guan, Min Zhang, Xiaolan Liu, Jing Li, Beibei Xin, Yanxin Ren, Yuchao Yang, Hui Wang, Mengjing Zhao, Yunpeng Huang, Xiaojing Guo, Jun Du, Wenbin Qian, Liping Su

**Affiliations:** ^1^ Shanxi Province Cancer Hospital/Shanxi Hospital Affiliated to Cancer Hospital, Chinese Academy of Medical Sciences/Cancer Hospital Affiliated to Shanxi Medical University, Taiyuan, China; ^2^ Department of Pathology, Shanxi Cancer Hospital, Affiliated Cancer Hospital of Shanxi Medical University, Taiyuan, China; ^3^ Department of Medicine, Shanghai Yuanqi Biomedical Technology Co., Ltd., Shanghai, China; ^4^ Department of Technology, Shanghai Yuanqi Biomedical Technology Co., Ltd., Shanghai, China; ^5^ Department of Biochemistry and Molecular Biology, School of Basic Medical Sciences, Shanxi Medical University, Taiyuan, China; ^6^ Department of Microbial and Biochemical Pharmacy, School of Pharmaceutical Sciences, Sun Yat-sen University, Guangzhou, China; ^7^ Department of Hematology, The Second Affiliated Hospital, College of Medicine, Zhejiang University, Hangzhou, China

**Keywords:** diffuse large B cell lymphoma, circulating tumor DNA, targeted next-generation sequencing, mutation, prognosis

## Abstract

**Background:**

Characterization of gene mutation profiles can provide new treatment options for patients with diffuse large B-cell lymphoma (DLBCL). However, this method is challenged by the limited source of tissue specimens, especially those of DLBCL patients at advanced stages. Therefore, in the current study, we aimed to describe the gene mutation landscape of DLBCL using circulating tumor DNA (ctDNA) samples obtained from patients’ blood samples, as well as to explore the relationship between ctDNA mutations and the prognosis and treatment response of patients with newly diagnosed DLBCL.

**Methods:**

A total of 169 newly diagnosed Chinese DLBCL patients were included in this study, among which 85 patients were divided into a training set and 84 were assigned into a validation set. The mutation profile of a 59-gene panel was analyzed by targeted next generation sequencing (NGS) of the patients’ ctDNA samples. Differences in clinical factors between patients with and without ctDNA mutations were analyzed. In addition, we also explored gene mutation frequencies between GCB and non-GCB subtypes, and the relationship between gene mutation status, clinical factors, mean VAF (variant allele frequencies) and the patients’ overall survival (OS) and progression-free survival (PFS).

**Results:**

ctDNA mutations were detected in 64 (75.3%) patients of the training set and 67 (79.8%) patients of the validation set. The most commonly mutated genes in both sets were *PCLO*, *PIM1*, *MYD88*, *TP53*, *KMT2D*, *CD79B*, *HIST1H1E* and *LRP1B*, with mutation frequencies of >10%. Patients with detectable ctDNA mutations trended to present advanced Ann Arbor stages (III-IV), elevated LDH (lactate dehydrogenase) levels, shorter OS and PFS, and a lower complete response (CR) rate to the R-CHOP regimen compared with DLBCL patients without ctDNA mutations. In addition, mean VAF (≥4.94%) and *PCLO* mutations were associated with poor OS and PFS.

**Conclusion:**

We investigated the ctDNA mutation landscape in Chinese patients with newly diagnosed DLBCL and found that ctDNA could reflect tumor burden and patients with detectable ctDNA mutations trended to have shorter OS and PFS and a lower CR rate.

## Background

Diffuse large B-cell lymphoma (DLBCL) is the most common type of non-Hodgkin’s lymphoma (NHL) worldwide with high clinical and genetic heterogeneity and worse outcomes (1). Gene expression profiling (GEP) divides DLBCL into two main subtypes, namely the germinal center B-cell (GCB) and activated B-cell (ABC) subtypes, with different responses to chemotherapy and targeted agents (2, 3). Recently, Schmitz et al. (4) and Wright et al. (5) classified DLBCL into five and seven genetic subtypes based on gene mutation and translocation profiles. Although these current genotyping techniques are widely accepted, they are challenged by the limited source of tissue specimens, especially for the detection of minimal residual disease (MRD). Thus, it is vital to develop alternative genotyping methods based on patients’ body fluids.

Liquid biopsy is a non-invasive method reflecting intra-tumor heterogeneity with no need for fresh tissues (6) and has potential values in diagnosis, MRD monitoring and treatment choice of lymphomas (7, 8). Circulating tumor DNA (ctDNA) is the DNA fragment derived from tumor cells, which accounts for about 0.1% of cell-free DNA (cfDNA) and emerges as one of the most powerful tools for the early diagnosis of cancers (9). Evidence has demonstrated that the allele frequencies (AFs) of individual mutations detected in tumor samples are highly correlated with those observed in paired plasma cfDNA samples (8, 10). Thus, an analysis of ctDNA in cancer patients can reveal both genetic alterations, including single nucleotide variants (SNVs), insertions/deletions (Indels), chromosomal rearrangements, and copy number variations (CNVs), which can be used for genotyping, and ctDNA content, which can reflect tumor burden (11). Kurtz et al. (11) explored the prognostic value of ctDNA level before and during immunochemotherapy for patients with DLBCL from North America and Europe; they found that pretreatment ctDNA level was an independent prognostic factor in DLBCL. Liu et al. (10) explored the mutation profiles in Chinese patients with newly diagnosed and relapsed/refractory (R/R) DLBCL and observed highly consistent ctDNA and tissue mutation profiles in these patients (sensitivity: 87.50%).

Considering that different races can have varied gene mutation profiles and that the clinical value of ctDNA in Chinese patients remains largely unknown, in this study, we explored the clinical significance of ctDNA in 169 newly diagnosed Chinese DLBCL patients. These patients were first divided into a training set and a validation set. Then we assessed the relationship between ctDNA mutations and clinicopathological features, as well as the roles of ctDNA mutations, including the detected mutation site/gene number, the mean VAF (variant allele frequency) and the mutation status of genes, in the overall survival (OS) and progression-free survival (PFS) in these patients.

## Patients and methods

### Patients

A total of 169 newly diagnosed Chinese DLBCL patients were enrolled at Shanxi Cancer Hospital from June 2018 to December 2019. Patients were considered eligible for inclusion if they aged ≥ 18 years and had histologically confirmed DLBCL according to the 2016 World Health Organization (WHO) Classification of Tumors of Haematopoietic and Lymphoid Tissues (12). The patients were classified into GCB and non-GCB subgroups according to the Hans algorithm (13). The disease was staged based on the 2014 Lugano Classification and the international prognositic index (IPI) was applied for risk stratification. Bone marrow involvement was assessed by flow cytometry, combined with immunoglobulin (Ig) gene rearrangement and positron emission tomography-computed tomography (PET-CT). A tumor lesion was judged as a bulky disease if the product of length and width of the tumor was ≥ 7.5 cm. All the patients were treated with the R-CHOP regimen (rituximab, cyclophosphamide, doxorubicin, vindesine, prednisone) and followed up until March, 2022. The treatment response, including CR (complete response), PR (partial response), SD (stable disease) and PD (progression disease), was assessed by CT/magnetic resonance imaging (MRI) and PET/CT according to the 2022 Guidelines of Chinese Society of Clinical Oncology (CSCO) after two to four cycles of the R-CHOP regimen. The data described in this manuscript were approved by the Ethics Committee of Shanxi Cancer Hospital (Ethical approval No.2021013) and conducted in accordance with the Helsinki declaration.

All study activities were approved by the Ethics Committee of Shanxi Cancer Hospital (Ethical approval No.2021013), and informed consent was obtained in accordance with the Declaration of Helsinki.

### DNA extraction and targeted sequencing

Ten milimeter of peripheral blood samples were collected using EDTA-containing tubes within 1 week of receiving anticancer treatment and centrifuged at 820 g for 10 min to obtain plasma samples, which were centrifuged at 20,000 g for 10 min. Next, cfDNA was extracted using the QIAamp Circulating Nucleic Acid Kit (QIAGEN, Gemany) according to the manufacturer’s instructions. Subsequently, the mutation profile of a 59-gene panel based on literatures (8, 14) was analyzed by targeted next generation sequencing (NGS) of the cfDNA samples (Shanghai Rightongene Bio-tech Co. Ltd, Shanghai, China; **Supplementary Table 1**) with Illumina NovaSeq 5000 (2×150-bp paired-end sequencing). In this study, VAF was defined as the ratio of the number of mutated alleles to the total number of alleles detected by NGS at a specific genome locus. Mutations with a VAF value ranging from 45% to 55% and ≥ 95% were identified and considered as heterozygous and homozygous germline mutations, respectively. Mean VAF was calculated as follows: Mean VAF = The sum of VAF values of all mutations/the total number of mutations.

### Statistical analysis

The maftools (“clinical Enrichment”) package of R was used to analyze the differences in clinical factors and gene mutation frequencies between the GCB and non-GCB subgroups using Chi-square test or Fisher’s exact test. The tableone package of R was applied to analyze the differences in mean VAF between the two groups. Survival probabilities were estimated using the Kaplan-Meier method. We considered two survival endpoints: PFS, the time intervals from diagnosis to progression, relapse, or death from any cause; and OS, the time intervals from diagnosis to death resulting from any cause. Factors with a P value <0.1 were included in the multivariate Cox regression models. P values < 0.05 were considered as statistically significant.

## Results

### Relationship between clinicopathological features and ctDNA mutation status in patients with newly diagnosed DLBCL

A total of 169 newly diagnosed DLBCL cases with valid targeted NGS data were included in this study, with 85 patients in the training set and 84 in the validation set. Detailed clinical information of the 169 patients is provided in **Supplementary Table 2**. Sixty-four (75.3%) patients of the training set carried ctDNA mutations. These patients were significantly enriched in Ann Arbor stages III-IV (69.8% vs. 38.1% in Ann Arbor stages I-II, P=0.002) and tended to have elevated LDH (lactic dehydrogenase) levels (53.1% vs. 14.3%, P=0.004) as compared with the patients without detectable ctDNA mutations (**Table 1**). Similar results were observed in the validation set, in which 67 (79.8%) patients having detectable ctDNA mutations were significantly enriched in advanced Ann Arbor stages (76.1% vs. 47.1%, P=0.041) and exhibited elevated LDH levels (56.7% vs. 18.8%, P=0.014) as compared with those without detectable ctDNA mutations (**Table 2**). In addition, the presence of ctDNA mutations was also associated with a higher incidence of bulky disease (41.8% vs. 0.0%, P=0.003) only in the validation group (**Table 2**). These results indicated that ctDNA mutation status was closely associated with the staging and LDH level of newly diagnosed DLBCL patients.

**Table 1:** The clinicopathologic features of DLBCL patients with mutation or without in training group.

Clinicopathologic features	Non-mutation group	Mutation group	*P*
Age, n (%)			0.663
≤60	10 (47.6)	25 (39.1)	
>60	11 (52.4)	39 (60.9)	
Gender			1.000
Female	8 (38.1)	24 (37.5)	
Male	13 (61.9)	40 (62.5)	
Bone marrow involvement, n (%)			0.192
No	21 (100.0)	54 (87.1)	
Yes	0 (0.0)	8 (12.9)	
Bulky disease, n (%)			0.131
No	19 (90.5)	44 (71.0)	
Yes	2 (9.5)	18 (29.0)	
Ann Arbor stage, n (%)			0.002
I-II	13 (61.9)	19 (30.2)	
II-IV	8 (38.1)	44 (69.8)	
Han’s classification, n (%)			0.319
GCB	9 (45.0)	19 (29.7)	
Non-GCB	11 (55.0)	45 (70.3)	
IPI score, n (%)			0.103
0-3	20 (95.2)	47 (75.8)	
4-5	1 (4.8)	15 (24.2)	
LDH, n (%)			0.004
Normal	18 (85.7)	30 (46.9)	
Elevated	3 (14.3)	34 (53.1)	
Response to therapy, n (%)			0.048
CR	14 (20.3)	26 (37.7)	
PR+SD+PD	4 (5.8)	25 (36.2)	

LDH, lactic dehydrogenase; CR, complete response; PR, partial response; SD, stable disease; PD, progression disease.

**Table 2:** The clinicopathologic features of DLBCL patients with mutation or without in validation group.

Clinicopathologic features	Non-mutation group	Mutation group	*P*
Age, n (%)			0.029
≤60	14 (82.4)	33 (49.3)	
>60	3 (17.6)	34 (50.7)	
Gender, n (%)			1.000
Female	7 (41.2)	29 (43.3)	
Male	10 (58.8)	38 (56.7)	
Bone marrow involvement, n (%)			0.525
No	17 (100.0)	58 (92.1)	
Yes	0 (0.0)	5 (7.9)	
Bulky disease			0.003
No	17 (100.0)	39 (58.2)	
Yes	0 (0.0)	28 (41.8)	
Ann Arbor stage, n (%)			0.041
I-II	9 (52.9)	16 (23.9)	
III-IV	8 (47.1)	51 (76.1)	
Han’s classification, n (%)			0.085
GCB	9 (52.9)	18 (27.3)	
Non-GCB	8 (47.1)	48 (72.7)	
IPI score, n (%)			0.058
0-3	17 (100.0)	51 (76.1)	
4-5	0 (0.0)	16 (23.9)	
LDH, n (%)			0.014
Normal	13 (81.2)	29 (43.3)	
Elevated	3 (18.8)	38 (56.7)	
Response to therapy, n (%)			
CR	12 (16.9)	26 (36.6)	
PR+SD+PD	4 (5.6)	29 (40.8)	

LDH, lactic dehydrogenase; CR, complete response; PR, partial response; SD, stable disease; PD, progression disease.

### ctDNA mutation profiles of patients with newly diagnosed DLBCL

**Figure 1:**
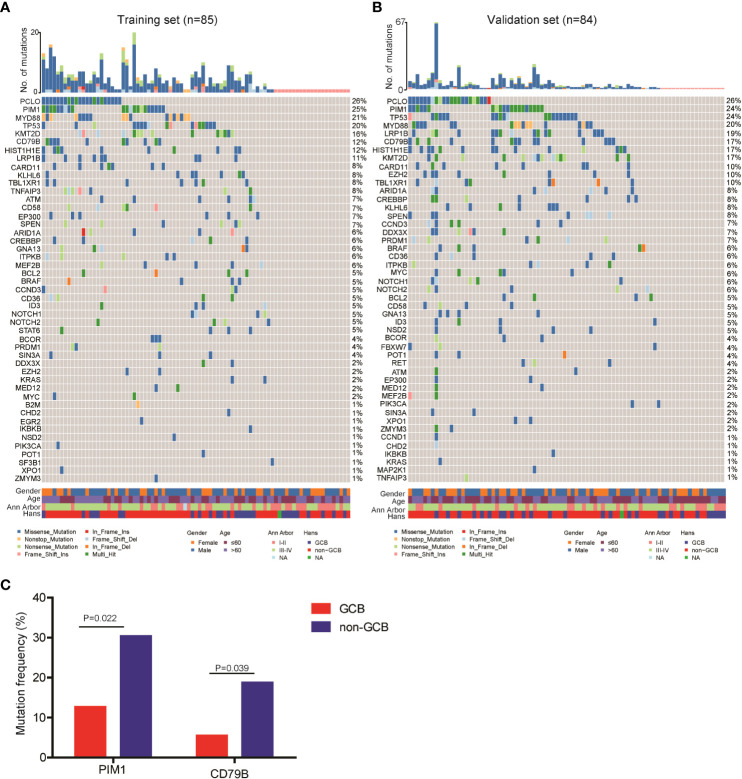
Mutation landscape of ctDNA samples from newly diagnosed DLBCL patients. ctDNA mutation profiles of newly diagnosed DLBCL patients in the **(A)** training and **(B)** validation sets. **(C)** The mutation frequencies of *PIM1* and *CD79B* were significantly increased in the ctDNA samples of non-GCB DLBCL patients as compared with GCB DLBCL patients.

Next, we explored the ctDNA mutation profiles of DLBCL patients of the training and validation sets. Detailed information on mutation sites is provided in **Supplementary Table 3** and **Supplementary Table 4**. On average, we detected 6.1 ± 7.1 genetic mutations in patients with confirmed ctDNA mutation. Mutations in *PCLO* (26%), *PIM1* (25%), *MYD88* (21%), *TP53* (20%), *KMT2D* (16%), *CD79B* (12%), *HIST1H1E* (12%) and *LRP1B* (11%) genes were the most frequently detected variations, with mutations in each of the genes being found in no less than 9 patients (10%) of the training set (**Figure 1A**). Consistently, *PCLO* (26%), *PIM1* (24%), *MYD88* (20%), *TP53* (24%), *KMT2D* (17%), *CD79B* (17%), *HIST1H1E* (17%) and *LRP1B* (20%) genes also showed high mutation frequencies in the validation set (**Figure 1B**). In addition, we assessed the difference in ctDNA mutation profile between the GCB and non-GCB subtypes in the 169 DLBCL patients. The results demonstrated that the mutation frequencies of *PIM1* (30.4% vs. 12.7%) and *CD79B* (18.8% vs. 5.5%) were significantly higher in patients of the non-GCB subtype than in those of the GCB subtype (**Figure 1C**). These results depicted the ctDNA mutation landscape of patients with newly diagnosed DLBCL.

### ctDNA mutation status was associated with the response to R-CHOP and clinical manifestation in newly diagnosed DLBCL patients

**Figure 2:**
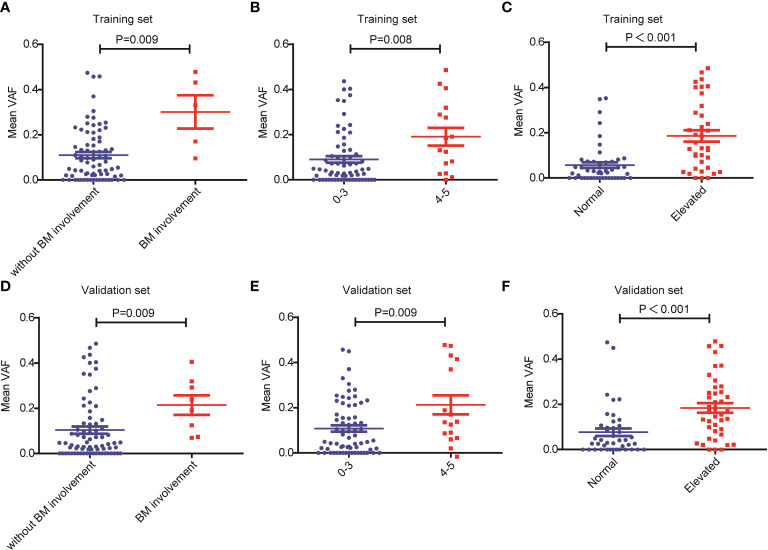
Relationship between mean VAF and clinical features in patients with newly diagnosed DLBCL. Mean VAF was increased in patients with **(A, D)** bone marrow involvement, **(B, E)** higher IPI scores and **(C, F)** elevated LDH levels in the training and validation sets.

Next, we assessed the relationship between ctDNA mutation status and the response to R-CHOP regimen in newly diagnosed DLBCL patients. The CR rate in DLBCL patients without ctDNA mutations was obviously higher than that in those carrying ctDNA mutations in both the training (P=0.048) and validation sets (P=0.050) (**Tables 1**, **2**). However, there were no valid differences in the rates of PR, SD and PD between DLBCL patients with different mutation numbers, which is mean VAF values and mutation profiles because the training and validation sets exhibited inconsistent findings. In addition, we compared the mean VAF value in patients with different ages (≤60 vs. >60 years), genders (male vs. female), bone marrow involvement statuses (positive vs. negative), Hans classifications (GCB vs. non-GCB), bulky disease statuses (positive vs. negative), IPIs (1-3 vs. 4-5), Ann Arbor stages (I-II vs. III-IV) and LDH levels (high vs. low). The results showed that the mean VAF value was significantly increased in patients with bone marrow involvement, higher IPI scores (4, 5) and elevated LDH levels in both of the training (**Figures 2A–C**) and validation sets (**Figures 2D–F**). Collectively, these results demonstrated that ctDNA mutations were associated with a lower CR rate and aggressive clinical manifestation in patients with newly diagnosed DLBCL.

### DLBCL patients carrying ctDNA mutations demonstrated poor prognosis

**Figure 3:**
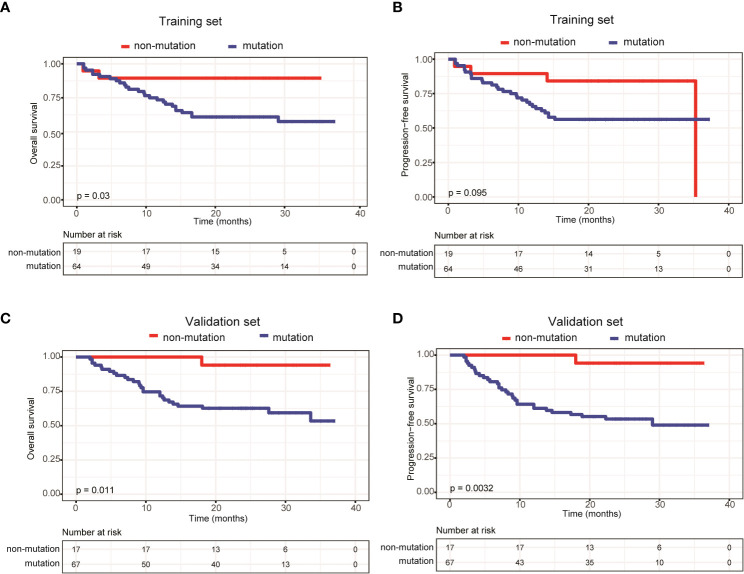
Patients carrying ctDNA mutations trended to have poor prognosis. Kaplan-Meier curves were applied to compare the **(A)** OS and **(B)** PFS between patients with and without ctDNA mutations in the training set. Kaplan-Meier curves were applied to compare the **(C)** OS and **(D)** PFS between patients with and without ctDNA mutations in the validation set.

We also compared survival outcomes between patients with and without ctDNA mutations. In the training set, the 64 patients with ctDNA mutations exhibited significantly shorter OS than the 19 patients without ctDNA mutations (P=0.03) (**Figure 3A**). PFS was also shorter in patients with ctDNA mutations, albeit the difference was not statistically significant (P=0.095) (**Figure 3B**). Next, we validated these results in the validation set, in which 67 patients had detectable ctDNA mutations and 17 patients did not. Compared with patients without ctDNA mutations, both OS (P=0.011) (**Figure 3C**) and PFS (P=0.0032) (**Figure 3D**) were significantly shorter in patients with ctDNA mutations. These results indicated that ctDNA mutations were associated with poor prognosis in patients with newly diagnosed DLBCL.

### Mean VAF and *PCLO* mutations were associated with poor prognosis in patients with newly diagnosed DLBCL

**Figure 4:**
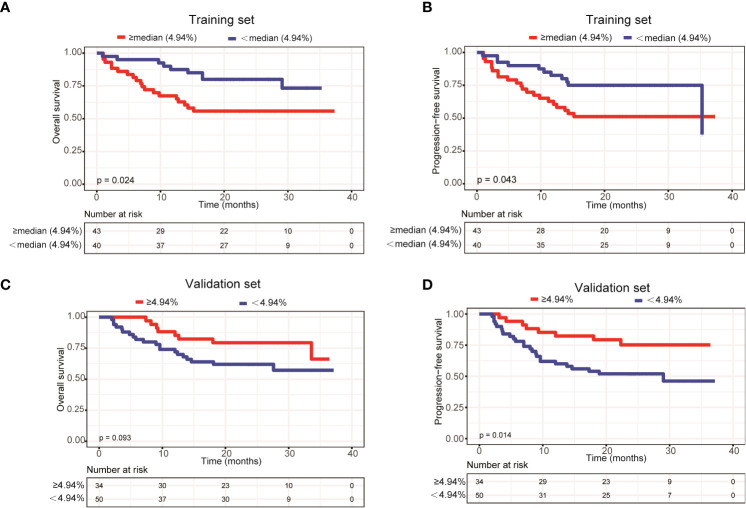
Patients with a mean VAF value ≥ 4.94% trended to have poor prognosis. Kaplan-Meier curves were applied to compare the **(A)** OS and **(B)** PFS between patients with mean VAF values ≥ 4.94% and < 4.94% in the training set. Kaplan-Meier curves were applied to compare the **(C)** OS and **(D)** PFS between patients with mean VAF values ≥ 4.94% and < 4.94% in the validation set.

To further explore the relationship between ctDNA mutation status and the prognosis of patients with newly diagnosed DLBCL, we assessed the effects of mutation number, mutated gene number and mean VAF on OS and PFS using Kaplan-Meier curves generated based the parameters’ average/median values. The results demonstrated that only mean VAF (the median value of which was 4.94%) was closely associated with patients’ prognosis in the training set. Specifically, the OS (P=0.024) and PFS (P=0.043) of patients with a mean VAF ≥ 4.94% were significantly shorter than those of patients with a mean VAF < 4.94% in the training set **(** **Figures 4A, B**). We next verified these findings in the validation set. Compared with those in patients with a mean VAF < 4.94%, the OS (P=0.093) and PFS (P=0.014) were shorter in patients with a mean VAF ≥ 4.94%, albeit the difference in OS was not statistically significant (**Figures 4C, D**).

**Figure 5:**
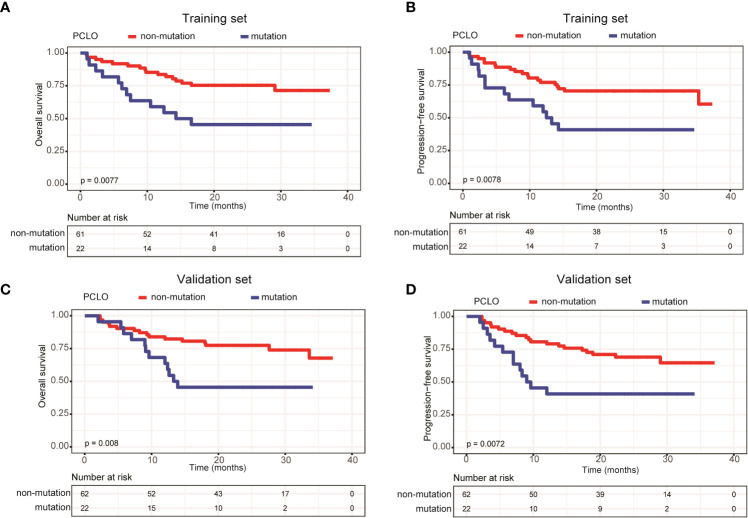
Patients carrying *PCLO* mutations trended to have poor prognosis. Kaplan-Meier curves were applied to compare the **(A)** OS and **(B)** PFS between patients with and without *PCLO* mutations in the training set. Kaplan-Meier curves were applied to compare the **(C)** OS and **(D)** PFS between patients with and without *PCLO* mutations in the validation set.

In addition, we assessed the effects of gene mutation status on the OS and PFS of patients with newly diagnosed DLBCL. Due to the relatively small sample size, we only assessed genes with a mutation frequency ≥ 10%. In the training set, *LRP1B* (**Supplementary Figures 1A, B**) and *PCLO* mutations (**Figures 5A, B**) were significantly associated with shorter OS and PFS; whereas in the validation set, only *PCLO* mutations were significantly associated with shorter OS and PFS (**Figures 5C, D**). These results demonstrated that a high mean VAF value and *PCLO* mutations predicted poor prognosis in patients with newly diagnosed DLBCL.

### Multivariate analysis of prognostic factors in patients with newly diagnosed DLBCL

Finally, multivariate Cox analysis was performed to further explore prognostic factors in patients with newly diagnosed DLBCL. The univariate Cox analysis showed that age > 60 years was an influencing factor on both OS (P=0.038) and PFS (P=0.083) in the training set; meanwhile, bulky disease status (P=0.099) was an influencing factor on PFS in the training set (**Table 3**). Afterwards, factors with a P value < 0.1, namely the clinical factors (age and/or bulky disease status), mean VAF, and *PCLO* mutation status, were included in the multivariate analysis. The results showed that age (> 60 years) and mean VAF (≥ 4.94%) were independent influencing factors on both OS and PFS in the training set (**Table 4**). In the validation set, age (> 60 years) and *PCLO* mutation status were influencing factors on OS, while age (> 60 years) and bulky disease status were influencing factors on PFS (**Table 4**) . These results further verified the close relationship between ctDNA mutation and the prognosis of patients with newly diagnosed DLBCL.

**Table 3:** Univariate Cox analysis of the influencing factors of PFS and OS in the training set.

Clinical factors	OS		PFS	
	HR (95% CI)	P	HR (95% CI)	P
Age (>60)	2.651 (1.053-6.671)	0.038	2.066 (0.909-4.694)	0.083
Ann Arbor stage (III-IV)	1.673 (0.697-4.013)	0.249	1.583 (0.697, 3.594)	0.273
Han’s classification (non-GCB)	0.710 (0.314-1.607)	0.411	0.870 (0.394-1.924)	0.731
Bulky disease	1.920 (0.846-4.360)	1.920	1.918 (0.885-4.159)	0.099
LDH level (elevated)	1.760 (0.799-3.881)	0.161	1.699 (0.808-3.572)	0.162

HR, Hazard ratio; CI, confidence interval.

**Table 4:** Multivariate Cox analysis of the influencing factors of PFS and OS in the training set and validation set.

Clinical factors	Training set				Validation set			
	OS		PFS		OS		PFS	
	HR (95% CI)	P	HR (95% CI)	P	HR (95% CI)	P	HR (95% CI)	P
Age (>60)	3.6 (1.4-9.2)	0.01	2.6 (1.1-6.4)	0.03	2.2 (1.0-4.9)	0.05	2.1 (1.0-4.4)	0.04
Bulky disease	—	—	1.2 (0.5-2.7)	0.69	—	—	2.4 (1.1-5.0)	0.03
VAF (4.94%)	2.6 (1.1-6.4)	0.04	2.5 (1.1-5.8)	0.04	1.4 (0.6-3.5)	0.51	1.4 (0.6-3.6)	0.48
*PCLO* mutation	1.9 (0.8-4.3)	0.15	1.8 (0.8-4.0)	0.17	2.7 (1.2-4.3)	0.02	2.0 (1.0-4.3)	0.06

HR, Hazard ratio; CI, confidence interval.

## Discussion

Genetic heterogeneity is a major cause of increased risk and treatment failure in DLBCL. Several studies (8, 10, 14–16) have proved that the mutations detected in blood samples were similar to those identified in tumor tissue, with a concordance rate over 80%.In the present study, we performed targeted sequencing of 59 lymphoma-related genes, the same panel as Liu et al. (10) to analyze the clinical value of ctDNA mutation in 169 Chinese patients with newly diagnosed DLBCL. To increase the reliability of our findings, the 169 patients were randomly divided into a training set (n=85) and a validation set (n=84). Our results demonstrated that detectable ctDNA mutations, a mean VAF value ≥ 4.94%, and *PCLO* mutations were strongly associated with shorter OS and PFS in the newly diagnosed DLBCL patients.

We found that *PCLO* (piccolo presynaptic cytomatrix protein), *PIM1, CD79B* and *MYD88* (genes involved in the NF-κB signaling pathway), *LRP1B* and *TP53* (tumor suppressive genes), as well as *KMT2D* and *HIST1H1E* (histone modifying genes) were the most commonly mutated genes in the 169 newly diagnosed DLBCL patients. According to the genetic landscape of DLBCL in western countries, the most frequently mutated genes are sequentially *KMT2D*, *MYD88*, *CREBBP*, *TP53* and *PIM1* (17, 18). In contract, the most frequently mutated genes in Chinese DLBCL patients are sequentially *PIM1*, *BTG2*, *TP53*, *HIST1H1E* and *KMT2D* (19). The higher proportion of non-GCB DLBCL cases in Chinese patients may be a reason for this difference. According to literature, genes related to histone methylation or acetylation (*EZH2*, *EP300*, *CREBBP* and *KMT2D*) and the PI3K/AKT and JAK/STAT pathways are commonly mutated in the GCB subtype of DLBCL patients, while genes related to the B-cell receptor and NF-κB signaling pathways, such as *MYD88*, *CD79A/B*, *CARD11*, *PIM1* and *TNFAIP3*, are commonly mutated in the ABC subtype (20). Consistently, we found that the mutation frequencies of *PIM1* and *CD79B* were significantly higher in DLBCL patients with the non-GCB subtype than in those with the GCB subtype.

In addition, we were able to detect ctDNA mutations in 64 (75.3%) out of 85 patients in the training set and 67 (79.8%) out of 84 patients in the validation set. Rivas-Delgado et al. (21) were able to detect at least one ctDNA mutation in 69 of 79 patients (87%) with DLBCL. This slight difference in ctDNA mutation detection rate may be caused by different panels of genes sequenced: Rivas-Delgado et al. (21) performed targeted sequencing on 112 genes, while we analyzed 59 genes. In addition, we found that patients with detectable ctDNA mutations had shorter OS and PFS in both the training and validation sets. Furthermore, patients carrying ctDNA mutations were significantly enriched in more advanced Ann Arbor stages (stages III-IV) and generally exhibited elevated LDH levels. These findings establish a link between ctDNA mutation status and the prognosis of patients with DLBCL. Recently, Kurtz et al. (22) indicated that 25% of ctDNA-negative patients demonstrated by cancer personalized profiling by deep sequencing (CAPP-Seq) were found to be ctDNA-positive, as revealed by phased variant enrichment and detection sequencing (PhasED-Seq), after two cycles of therapy and presented with poor outcomes.

ctDNA VAF has been closely associated with the clinical features and prognosis of various cancers, and is considered as a new biomarker for tumor burden (23, 24). For example, Fu et al. (25) found that the VAF values of *TP53* p.Y88C and *LATS2* p.F972L were decreased in B-cell lymphoma patients with CR. Desch et al. (26) reported that ctDNA VAF values were strongly associated with total metabolic tumor volume (TMTV) and the incidence of bulky disease in pediatric Hodgkin’s lymphoma. In addition. the median VAF of non-DNMT3A clones increased from 1% at the time of autologous stem cell transplantation (ASCT) to 37% at the diagnosis of therapy-related myeloid neoplasms (tMNs) (27). In the present study, we found that the mean VAF values were significantly increased in patients with bone marrow involvement, higher IPI scores and elevated LDH levels in both the training and validation sets. Additionally, we observed that in the training set, patients with a mean VAF ≥ 4.94% showed inferior OS and PFS as compared with patients with a mean VAF < 4.94%. This finding was verified in the validation set.

Moreover, we assessed the relationship between ctDNA mutation status and the prognosis of patients with newly diagnosed DLBCL. Notably, we found that patients with *PCLO* mutations had shorter OS and PFS. *PCLO* encodes a protein that functions as a part of the presynaptic cytoskeletal matrix, which is thought to be involved in neurotransmitter release regulation. It has been suggested that *PCLO* might play a role in calcium sensing. *PCLO* mutations have been detected by whole-exom sequencing in a variety of tumors, including DLBCL (28–31). In the mesenchymal subtype of glioblastomas, *PCLO* mutations have been shown to be associated with poor prognosis (31), but its association with the prognosis of DLBCL has not been reported. Mutations in *PCLO* are usually considered as passenger mutations with no functional consequences in DLBCL (28). In this study, *PIM1* (34.1%), *MYD88* (31.8%) and *TP53* (20.5%) were the most common co-mutated genes with *PCLO* mutations detected in the ctDNA samples of DLBCL patients. Furthermore, we found that the mutation frequency of *TNFAIP3* in *PCLO* mutated DLBCL patients was significantly higher than that of DLBCL patients without *PCLO* mutations [1.6% (2/125) vs. 13.6% (6/44)]. These four genes (*PIM1*, *MYD88*, *TP53, TNFAIP3*) has been identified to be the mutational drivers in DLBCL, which might partly explain the poor prognosis of patients carrying *PCLO* mutations (32–35). Additional work is needed to resolve the mechanism of action and role of *PCLO* mutations in DLBCL.

Evidence has demonstrated that ctDNA mutations are correlated with treatment response in DLBCL patients (36). According to the current gold standard for evaluating treatment response in lymphoma, the sensitivity and specificity of ctDNA profiling were 94.7% and 83.3% in refractory or relapse (r/r) DLBCL patients after CAR-T treatment; the median numbers of baseline ctDNA mutations in patients who remained long-term CR and in patients who relapsed or became refractory to CAR-T therapy were 3.0 and 14.3, respectively (36). Herein, we explored the relationship between ctDNA mutation status, the number of ctDNA mutations and mean VAF and the curative effect of R-CHOP regimen in DLBCL patients. Our results showed that patients without detectable ctDNA mutations had a higher CR rate to R-CHOP treatment as compared with patients with detectable ctDNA mutations, while the ctDNA mutation number and mean VAF showed no significant impacts on the CR rate.

Our study showed that age (> 60 years) and mean VAF (≥ 4.94%) were independent influencing factors on prognosis in the training set, while age (> 60 years), *PCLO* mutations and bulky disease status were independent influencing factors on prognosis in the validation set. The high heterogeneity of DLBCL may have caused these differences between the training and validation sets. Of course, the small sample size of our study may be another reason for the differences. In fact, the relatively small sample size is the main limitation of the present study, although we have recruited the largest cohort of Chinese DLBCL patients to date. To this end, we intend to include more Chinese DLBCL patients for analysis in the future.

Taken together, we herein have described the ctDNA mutation landscape of a largest cohort of Chinese patients with newly diagnosed DLBCL to date. Our results suggested that patients with detectable ctDNA mutations, a higher mean VAF value or *PCLO* mutations trended to have shorter OS and PFS and a lower CR rate. Our study provides evidence to support the feasibility of using ctDNA samples obtained from patients’ blood in prognosis prediction of newly diagnosed DLBCL.

## Data availability statement

The data presented in the study are deposited in the National Genomics Data Center repository (https://ngdc.cncb.ac.cn), accession number PRJCA012539.

## Ethics statement

The studies involving human participants were reviewed and approved by The Ethics Committee of Shanxi Cancer Hospital. Written informed consent for participation was not required for this study in accordance with the national legislation and the institutional requirements.

## Author contributions

Concept and design: LS, WQ, JD. Acquisition, analysis, or interpretation of data: TG, MeZ, JL, XL, YR, YY, HW, XG, YH. Patients’ follow-up: MiZ, YY, MeZ. Statistical analysis: BX, TG, MJZ. Drafting of the manuscript: TG, MiZ, BX. Supervision: LS. All authors contributed to the article and approved the submitted version.

## Funding

This study was supported by the foundation of Key Laboratory Construction Project Supported by Health Commission of Shanxi Province (Grant No. 2020SYS11), Shanxi Province Health Research Project Supported by Health Commission of Shanxi Province (Grant No. 2022121), and Research Project Supported by Shanxi Scholarship Council of China (Grant No. 2020-194).

## Acknowledgments

We thank Shanghai Rightongene Biotechnology Co. Ltd. (Shanghai, China) for the assistance in targeted sequencing and mutation analysis. In the meanwhile, we sincerely thank Hongsheng Wang, Department of Tumor Pharmacology, SUN YAT-SEN UNIVERSITY, for his linguistic and logic advise.

## Conflict of interest

Author BX and YR are employed by Shanghai Yuanqi Biomedical Technology Co., Ltd.

The remaining authors declare that the research was conducted in the absence of any commercial or financial relationships that could be construed as a potential conflict of interest.

## Publisher’s note

All claims expressed in this article are solely those of the authors and do not necessarily represent those of their affiliated organizations, or those of the publisher, the editors and the reviewers. Any product that may be evaluated in this article, or claim that may be made by its manufacturer, is not guaranteed or endorsed by the publisher.

## References

[1] Coiffier B Thieblemont C Van Den Neste E Lepeu G Plantier I Castaigne S. Long-term outcome of patients in the lnh-98.5 trial, the first randomized study comparing rituximab-chop to standard chop chemotherapy in dlbcl patients: A study by the groupe d'etudes des lymphomes de l'adulte. Blood (2018) 2010(12):2040–5. 10.1182/blood-2010-03-276246 PMC2951853 20548096

[2] Alizadeh AA Eisen MB Davis RE Ma C Lossos IS Rosenwald A. Distinct types of diffuse Large b-cell lymphoma identified by gene expression profiling. Nature (2000) 403(6769):503–11. doi: 10.1038/35000501 10676951

[3] Rosenwald A Wright G Chan WC Connors JM Campo E Fisher RI. The use of molecular profiling to predict survival after chemotherapy for diffuse Large-B-Cell lymphoma. N Engl J Med (2002) 346(25):1937–47. doi: 10.1056/NEJMoa012914 12075054

[4] Schmitz R Wright GW Huang DW Johnson CA Phelan JD Wang JQ. Genetics and pathogenesis of diffuse Large b-cell lymphoma. N Engl J Med (2018) 378(15):1396–407. doi: 10.1056/NEJMoa1801445 PMC6010183 29641966

[5] Wright GW Huang DW Phelan JD Coulibaly ZA Roulland S Young RM. A probabilistic classification tool for genetic subtypes of diffuse Large b cell lymphoma with therapeutic implications. Cancer Cell (2020) 37(4):551–68.e14. doi: 10.1016/j.ccell.2020.03.015 32289277 PMC8459709

[6] Decruyenaere P Offner F Vandesompele J. Circulating rna biomarkers in diffuse Large b-cell lymphoma: A systematic review. Exp Hematol Oncol (2021) 10(1):13. doi: 10.1186/s40164-021-00208-3 33593440 PMC7885416

[7] Cirillo M Craig AFM Borchmann S Kurtz DM. Liquid biopsy in lymphoma: Molecular methods and clinical applications. Cancer Treat Rev (2020) 91:102106. doi: 10.1016/j.ctrv.2020.102106 33049623 PMC8043056

[8] Rossi D Diop F Spaccarotella E Monti S Zanni M Rasi S. Diffuse Large b-cell lymphoma genotyping on the liquid biopsy. Blood (2017) 129(14):1947–57. doi: 10.1182/blood-2016-05-719641 28096087

[9] Cescon DW Bratman SV Chan SM Siu LL. Circulating tumor DNA and liquid biopsy in oncology. Nat Cancer (2020) 1(3):276–90. doi: 10.1038/s43018-020-0043-5 35122035

[10] Liu H Yang C Zhao X Le J Wu G Wei J. Genotyping on ctdna identifies shifts in mutation spectrum between newly diagnosed and Relapse/Refractory dlbcl. Onco Targets Ther (2020) 13:10797–806. doi: 10.2147/OTT.S275334 PMC7591234 33122918

[11] Kurtz DM Scherer F Jin MC Soo J Craig AFM Esfahani MS. Circulating tumor DNA measurements as early outcome predictors in diffuse Large b-cell lymphoma. J Clin Oncol (2018) 36(28):2845–53. doi: 10.1200/JCO.2018.78.5246 PMC6161832 30125215

[12] Swerdlow SH Campo E Pileri SA Harris NL Stein H Siebert R. The 2016 revision of the world health organization classification of lymphoid neoplasms. Blood (2016) 127(20):2375–90. doi: 10.1182/blood-2016-01-643569 PMC4874220 26980727

[13] Hans CP Weisenburger DD Greiner TC Gascoyne RD Delabie J Ott G. Confirmation of the molecular classification of diffuse Large b-cell lymphoma by immunohistochemistry using a tissue microarray. Blood (2004) 103(1):275–82. doi: 10.1182/blood-2003-05-1545 14504078

[14] Scherer F Kurtz DM Newman AM Stehr H Craig AF Esfahani MS. Distinct biological subtypes and patterns of genome evolution in lymphoma revealed by circulating tumor DNA. Sci Transl Med (2016) 8(364):364ra155. doi: 10.1126/scitranslmed.aai8545 PMC5490494 27831904

[15] Bohers E Viailly PJ Dubois S Bertrand P Maingonnat C Mareschal S. Somatic mutations of cell-free circulating DNA detected by next-generation sequencing reflect the genetic changes in both germinal center b-Cell-Like and activated b-Cell-Like diffuse Large b-cell lymphomas at the time of diagnosis. Haematologica (2015) 100(7):e280–4. doi: 10.3324/haematol.2015.123612 PMC4486242 25749829

[16] Camus V Sarafan-Vasseur N Bohers E Dubois S Mareschal S Bertrand P. Digital pcr for quantification of recurrent and potentially actionable somatic mutations in circulating free DNA from patients with diffuse Large b-cell lymphoma. Leuk Lymphoma (2016) 57(9):2171–9. doi: 10.3109/10428194.2016.1139703 26883583

[17] Karube K Enjuanes A Dlouhy I Jares P Martin-Garcia D Nadeu F. Integrating genomic alterations in diffuse Large b-cell lymphoma identifies new relevant pathways and potential therapeutic targets. Leukemia (2018) 32(3):675–84. doi: 10.1038/leu.2017.251 PMC5843901 28804123

[18] Reddy A Zhang J Davis NS Moffitt AB Love CL Waldrop A. Genetic and functional drivers of diffuse Large b cell lymphoma. Cell (2017) 171(2):481–94.e15. doi: 10.1016/j.cell.2017.09.027 28985567 PMC5659841

[19] Ren W Ye X Su H Li W Liu D Pirmoradian M. Genetic landscape of hepatitis b virus-associated diffuse Large b-cell lymphoma. Blood (2018) 131(24):2670–81. doi: 10.1182/blood-2017-11-817601 PMC6063049 29545328

[20] Pasqualucci L Dalla-Favera R. Genetics of diffuse Large b-cell lymphoma. Blood (2018) 131(21):2307–19. doi: 10.1182/blood-2017-11-764332 PMC5969374 29666115

[21] Rivas-Delgado A Nadeu F Enjuanes A Casanueva-Eliceiry S Mozas P Magnano L. Mutational landscape and tumor burden assessed by cell-free DNA in diffuse Large b-cell lymphoma in a population-based study. Clin Cancer Res (2021) 27(2):513–21. doi: 10.1158/1078-0432.CCR-20-2558 33122345

[22] Kurtz DM Soo J Co Ting Keh L Alig S Chabon JJ Sworder BJ. Enhanced detection of minimal residual disease by targeted sequencing of phased variants in circulating tumor DNA. Nat Biotechnol (2021) 39(12):1537–47. doi: 10.1038/s41587-021-00981-w PMC8678141 34294911

[23] van Velzen MJM Creemers A van den Ende T Schokker S Krausz S Reinten RJ. Circulating tumor DNA predicts outcome in metastatic gastroesophageal cancer. Gastric Cancer (2022) 25(5):906–15. doi: 10.1007/s10120-022-01313-w PMC9365750 35763187

[24] Arisi MF Dotan E Fernandez SV. Circulating tumor DNA in precision oncology and its applications in colorectal cancer. Int J Mol Sci (2022) 23(8):4441. doi: 10.3390/ijms23084441 35457259 PMC9024503

[25] Fu H Zhou H Qiu Y Wang J Ma Z Li H. Sept6_Trim33 gene fusion and mutated Tp53 pathway associate with unfavorable prognosis in patients with b-cell lymphomas. Front Oncol (2021) 11:765544. doi: 10.3389/fonc.2021.765544 34926267 PMC8671703

[26] Desch AK Hartung K Botzen A Brobeil A Rummel M Kurch L. Genotyping circulating tumor DNA of pediatric Hodgkin lymphoma. Leukemia (2020) 34(1):151–66. doi: 10.1038/s41375-019-0541-6 31431735

[27] Soerensen JF Aggerholm A Rosenberg CA Bill M Kerndrup GB Ebbesen LH. Clonal evolution in patients developing therapy-related myeloid neoplasms following autologous stem cell transplantation. Bone marrow Transplant (2022) 57(3):460–5. doi: 10.1038/s41409-022-01567-z 35027675

[28] Lohr JG Stojanov P Lawrence MS Auclair D Chapuy B Sougnez C. Discovery and prioritization of somatic mutations in diffuse Large b-cell lymphoma (Dlbcl) by whole-exome sequencing. Proc Natl Acad Sci U S A (2012) 109(10):3879–84. doi: 10.1073/pnas.1121343109 PMC3309757 22343534

[29] Wang H Shen L Li Y Lv J. Integrated characterisation of cancer genes identifies key molecular biomarkers in stomach adenocarcinoma. J Clin Pathol (2020) 73(9):579–86. doi: 10.1136/jclinpath-2019-206400 PMC7476269 32034058

[30] Qiu Z Lin A Li K Lin W Wang Q Wei T. A novel mutation panel for predicting etoposide resistance in small-cell lung cancer. Drug Des Devel Ther (2019) 13:2021–41. doi: 10.2147/DDDT.S205633 PMC6594009 31417239

[31] Park AK Kim P Ballester LY Esquenazi Y Zhao Z. Subtype-specific signaling pathways and genomic aberrations associated with prognosis of glioblastoma. Neuro Oncol (2019) 21(1):59–70. doi: 10.1093/neuonc/noy120 30053126 PMC6303485

[32] Juskevicius D Lorber T Gsponer J Perrina V Ruiz C Stenner-Liewen F. Distinct genetic evolution patterns of relapsing diffuse Large b-cell lymphoma revealed by genome-wide copy number aberration and targeted sequencing analysis. Leukemia (2016) 30(12):2385–95. doi: 10.1038/leu.2016.135 27198204

[33] Ngo VN Young RM Schmitz R Jhavar S Xiao W Lim KH. Oncogenically active Myd88 mutations in human lymphoma. Nature (2011) 470(7332):115–9. doi: 10.1038/nature09671 PMC5024568 21179087

[34] Lu TX Young KH Xu W Li JY. Tp53 dysfunction in diffuse Large b-cell lymphoma. Crit Rev Oncol Hematol (2016) 97:47–55. doi: 10.1016/j.critrevonc.2015.08.006 26315382

[35] Chapuy B Stewart C Dunford AJ Kim J Kamburov A Redd RA. Molecular subtypes of diffuse Large b cell lymphoma are associated with distinct pathogenic mechanisms and outcomes. Nat Med (2018) 24(5):679–90. doi: 10.1038/s41591-018-0016-8 PMC6613387 29713087

[36] Zhou L Zhao H Shao Y Chen X Hong R Wang L. Serial surveillance by circulating tumor DNA profiling after chimeric antigen receptor T therapy for the guidance of R/R diffuse Large b cell lymphoma precise treatment. J Cancer (2021) 12(18):5423–31. doi: 10.7150/jca.60390 PMC8364638 34405005

