# Supplementary figures and images for: Circulating tumor DNA mutation profile is associated with the prognosis and treatment response of Chinese patients with newly diagnosed diffuse large B-cell lymphoma
Source: Front Oncol. 2022 Nov 17;12:1003957. doi: 10.3389/fonc.2022.1003957 (PMC9713409; doi:10.3389/fonc.2022.1003957)

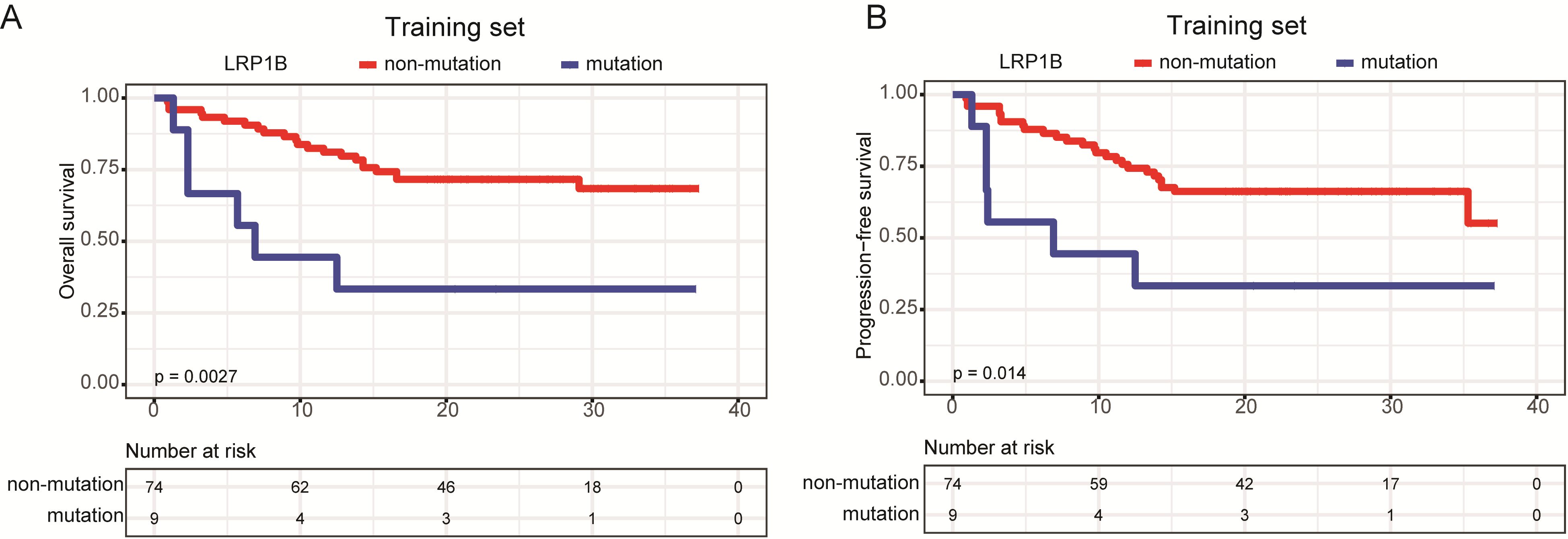

Supplement: Supplementary file 2 [file Image_1.tif]
